# Understanding PTSD in Portuguese Youth: Predictors and Risk Factors in a Multi‐Clinic, Treatment‐Engaged Sample

**DOI:** 10.1002/brb3.70805

**Published:** 2025-09-02

**Authors:** Inês Barroca, Inês Pinto, Paula Saraiva Carvalho

**Affiliations:** ^1^ Child and Adolescent Psychiatry Service Unidade Local de Saúde de Lisboa Ocidental Lisbon Portugal; ^2^ Faculty of Health Sciences Universidade da Beira Interior Covilhã Portugal; ^3^ Faculty of Medicine of Lisbon (FMUL) Lisbon Portugal; ^4^ Católica Medical School Lisbon Portugal; ^5^ ISPA (Superior Institute of Applied Psychology) Lisbon Portugal; ^6^ Child and Adolescent Psychiatry Service ULS Loures‐Odivelas Loures Portugal; ^7^ Department of Psychology and Education University of Beira Interior Covilhã Portugal; ^8^ Research Center in Sports Sciences Health Sciences and Human Development (CIDESD) Covilhã Portugal

**Keywords:** adolescents, children, predictors, PTSD, trauma

## Abstract

**Introduction:**

Posttraumatic stress disorder (PTSD) in childhood and adolescence is common. Studies have focused on a small group of predictors related to the traumatic event and still focus on the adult population.

**Objective:**

To explore the prevalence of PTSD and to identify factors that potentially increase the risk for the development of PTSD in a clinical sample of children and adolescents. Eligibility criteria included: experienced at least one traumatic event; age between 7 and 18 years; follow‐up period of at least 1 month. Data collection was achieved by using: clinical records to obtain the patients’ clinical data; the Clinician‐Administered PTSD Scale and the Checklist of Potentially Traumatic Events in Children and Adolescents.

**Results:**

A total of 101 participants were included. The prevalence of PTSD was 35.6%. For pre‐traumatic factors, significant association was found for age (*p* = 0.033), suggesting increased likelihood of PTSD for older participants. Regarding the type of event, PTSD was significantly associated with interpersonal events (*p* = 0.001). Participants who were a single intervenient (involved person) had increased odds for PTSD (*p* = 0.036). It was found that the association with PTSD, in a decreasing manner, occurred with dissociative symptoms, followed by symptoms of Group C (avoidance), Group B (intrusive thoughts), Group E (activation and reactivity) and Group D (cognitions and mood). Dissociative symptoms were significantly associated with PTSD (*p* = 0.001).

**Conclusion:**

The study provides evidence that several factors can predict the development of PTSD in childhood and adolescence. Awareness about these factors, healthcare workers’ specific training, and prevention and intervention strategies are the foundation to promote child well‐being throughout life.

## Graphical abstract

PTSD affected 35.6% of Portuguese youths in treatment. Older age, interpersonal trauma, being the sole intervenient, and dissociative symptoms significantly increased the risk of PTSD

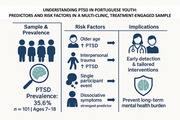

## 1 Introduction

From a historical perspective, the diagnosis of posttraumatic stress disorder (PTSD) was conceptualized in response to observations from the Vietnam War (Rutter 2015). Later, symptoms were also observed after exposure to other types of events, and it was realized that this condition could occur at any age (Rutter 2015; Charnsil et al. 2020). Although the original concept implies the rarity of this disorder, the literature supports a high rate of traumatic events in childhood and adolescence (Rutter 2015; Charnsil et al. 2020).

Although the development of PTSD occurs in a minority of cases, the prevalence of PTSD in children may be equal to or greater than that already known in the adult population (Rutter 2015; Charnsil et al. 2020; American Psychiatric Association 2000). Unlike the results stated in previous studies, which were based on the methodologies applied, PTSD rates can vary widely but appear to be approximately 15%–30% among children (Rutter 2015; Alisic et al. 2014; Mueser and Taub 2008). Accordingly, it is surmised that PTSD has been underdiagnosed, thus highlighting the importance of greater training in diagnosing it at a younger age and developing better and more effective assessment strategies (Rutter 2015).

Currently, according to the DSM‐5, this disorder no longer belongs to the group of anxiety disorders, but rather, it presents a set of characteristic symptoms that are divided into 4 groups: B—intrusive thoughts; C—avoidance; D—negative changes in cognitions and mood; and E—changes in activation and reactivity (American Psychiatric Association 2000). To make a diagnosis, symptoms must be present for at least 1 month and may or may not be associated with dissociative symptoms (American Psychiatric Association 2000). Although the general categorization is used, there are some particularities in the clinical presentation in childhood, which is why the DSM‐5‐TR also presents a list of symptoms that is better adapted for children under 6 years of age. However, it does not mention other differences throughout the rest of childhood and adolescence.

Some factors have been reported that may predict the development of PTSD. These factors are grouped into pre‐, peri‐, or post‐traumatic factors, depending on the moment of their occurrence. Some of these factors are related to the sociodemographic situation, the diversity of the type of event, the characteristics of the exposure, or the socio‐family support that occurs after the exposure (Rutter 2015; El‐Khodary et al. 2020; American Psychiatric Association 2013).

Despite the growing body of literature in this field, studies have focused on one or a small group of predictors and characteristics related to the exposure to a certain type of traumatic event. There is still little evidence on a complete assessment of various types of predictors in certain populations, which could provide a more complete view of the functioning of this pathology in certain populations. It should also be considered that the majority of studies focus on the adult population. To the best of our knowledge, no study has ever been conducted with respect to the Portuguese population with the purpose of assessing predictors of PTSD in childhood and adolescence.

Thus, the aim of this study was to explore the prevalence of PTSD, based on the DSM‐5 criteria, and identify factors that could be associated with the risk for the development of PTSD in a clinical sample of children and adolescents in Lisbon, Portugal.

## 2 Methods

### 2.1 Participants and Procedures

This was a multicenter, analytical cross‐sectional study on the prevalence and predictors of PTSD in children and adolescents conducted at the Units of Child Psychiatry or the Paediatric Clinic of Unidade Local de Saúde (ULS) de Lisboa Ocidental, ULS de Amadora/Sintra (formerly known as Hospital Fernando da Fonseca), and ULS de Loures‐Odivelas (formerly known as Hospital Beatriz Ângelo). In each center, the study was approved by the institutional ethics committee for clinical research.

The eligibility criteria were as follows: (1) experienced at least one traumatic event; (2) aged between 7 and 18 years; and (3) had a follow‐up period of at least 1 month.

The exclusion criteria were as follows: is not fluent in the Portuguese language, is cognitively impaired, or has a psychotic disorder.

Data collection was achieved by using clinical records to obtain the patients’ clinical data; the results of the Clinician‐Administered PTSD Scale, a semistructured scale to assess DSM‐5 criteria in children and adolescents (CAPS‐CA‐5) (Barroca et al. 2022); and the results of the clinical interviews using the Checklist of Potentially Traumatic Events in Children and Adolescents (CEPT‐CA) (Barroca et al. 2022).

To assess the predictors of PTSD, a 3‐group classification was used: pre‐, peri‐, and posttraumatic factors. The pre‐traumatic factors included sex, age at exposure to the event, previous exposure to traumatic events, family type (nuclear/extended; single‐parent; reconstructed; other), and the adapted Graffar index. The Graffar Index is a widely used instrument for assessing socioeconomic status (SES), and in its adapted version for the Portuguese population (Lima et al., 1985), it evaluates five dimensions: (1) profession of the family's main provider, (2) education level of the parents, (3) source of income, (4) housing conditions, and (5) neighborhood characteristics. Each item is scored on a scale from 1 to 5, with higher total scores indicating lower SES. Final scores are grouped into four socioeconomic classes: I (high), II (upper‐middle), III (middle), IV (lower‐middle), and V (low) (Lima et al. 1985).

The following peri‐traumatic factors were assessed: event type; exposure type (“It happened to me,” “I saw it happen to someone else,” “They told me”); perceived threat to life; serious injury to oneself or another; participants in the event (oneself, oneself and others, others); and symptoms (namely, dissociative symptoms).

Posttraumatic factors were assessed to determine whether there was subsequent exposure to adverse events.

### 2.2 Statistical Analysis

The first author introduced the clinical information and the data obtained from the administration of the CAPS‐CA‐5 and CEPT‐CA to each patient into the password‐protected project database. The information provided by the first author was confirmed by the last author.

Statistical analysis was performed using SPSS, version 29.0 (IBM Corp., Los Angeles, CA, USA, 2023). Means and standard deviations (SDs) were used to describe normally distributed continuous variables. Medians and quartiles (Q1‐Q3) were used to describe continuous nonnormally distributed variables. Normality was assessed using the Kolmogorov‒Smirnov test and by checking QQ plots. Categorical variables are described as frequencies and percentages. Comparisons of symptomatology by sex and age were performed with Mann‒Whitney tests. PTSD was assessed based on associations with all sample characteristics via logistic regressions. Effect sizes were calculated as odds ratios (ORs) and adjusted odds ratios (aORs) when adjusting for covariates. Confidence intervals and *p*‐values were used to determine significance. For all comparisons, statistical significance was assumed at *p* < 0.05.

## 3 Results

A total of 101 individuals participated during the study recruitment period.

### 3.1 Prevalence of PTSD

As defined by the CAPS‐CA‐5, the prevalence of PTSD was 35.6% (36/101), with late onset in 3.0% (3/101) of cases.

### 3.2 Pre‐Traumatic Factors

With respect to sex, 51.5% (52/101) were females, and 48.5% (49/101) were males. The age of exposure to the selected traumatic event varied between 3 and 17 years, with a mean of 10.7 years (SD = 3.4). The majority of the participants (64.4%) were children, that is, they ranged in age from 3 years to 12 years. The majority of participants had previous exposure to at least one traumatic event (71.3%). The majority of families were nuclear or extended (67.3%). The Graffar index was mostly ≥ 3 (with the majority being from Group 4, followed closely by Group 3).

### 3.3 Peri‐Traumatic Factors

With respect to the type of event, physical, verbal, and sexual violence events represented half of all events (51.5%). Regarding the type of exposure, 67.3% of the events happened to the individual, 15.8% witnessed the event, and 16.8% learned about the event. Approximately two‐thirds of the events were perceived as life‐threatening events (68.3%), whereas events that resulted in serious injuries represented 20.8% of all events. For 16.8% of the cases, exposure to the events occurred to oneself; for 46.5% of the cases, exposure to the events occurred to oneself or others; and for the remaining 36.6% of the cases, exposure to the events occurred through learning about it. The presence of dissociative symptoms occurred in 10.9% of the patients.

### 3.4 Posttraumatic Factors

The prevalence of subsequent exposure to adverse events was 66.3%. The majority of cases were related to physical, verbal, or sexual violence events and situations related to institutionalization processes. In several cases, this refers to the moment when child protection professionals — typically social workers and/or legal representatives from national child welfare services — appeared at the child's home without prior warning to enforce an institutionalization order, sometimes accompanied by police officers. These moments were frequently described by the children and families as highly distressing, often involving intense verbal conflict and, in some instances, physical tension. Although these professionals operate independently from the mental health clinics involved in the study, the psychological impact of such interventions was reported during clinical assessments and considered as a relevant posttraumatic factor in the analysis.

### 3.5 Associations Between Pre‐, Peri‐, and Posttraumatic Factors and the Development of PTSD

#### 3.5.1 Pre‐Traumatic Factors and PTSD

With respect to pre‐traumatic factors and PTSD, a significant association was found for age (OR = 1.15; *p* = 0.033), suggesting an increased likelihood of PTSD in older participants (Table Table 1 and 2). Sex, previous exposure to traumatic events, family type, and the Graffar index were not significantly associated with PTSD (Table 2).

**TABLE 1:** Pre‐, peri‐, and posttraumatic factors.

Pre‐Trauma	Peri‐Trauma	Post‐Trauma
Sex	Event type	Subsequent exposure to adverse events
Age of exposure to the event	Exposure type	
Previous exposure to traumatic events	Perceived threat to life	
Family type	Serious injury to yourself or another	
Socioeconomic Status (Graffar Index)	Participants	
	Symptoms	

**TABLE 2:** Pre‐traumatic factors and PTSD.

Pre‐Trauma				
	Sig.	Exp(B)	95% C.I. for EXP(B)	
			Lower	Upper
Sex	0.152	1.833	0.800	4.199
Age of exposure to the event	0.033*	1.149	1.011	1.304
Previous exposure to traumatic events	0.877	1.074	0.435	2.653
Family type	0.950	0.966	0.333	2.803
Graffar index	0.719	0.914	0.561	1.489

#### 3.5.2 Peri‐Traumatic Factors and PTSD

When sex and age were adjusted, PTSD was significantly associated with physical, verbal, or sexual violence events (aOR = 4.60, 95% CI = [1.83–11.61], *p* = 0.001) compared with other events. Participants who were a single intervenient (involved person) had increased odds for PTSD (aOR = 3.89, *p* = 0.036; Table 3).

**TABLE 3:** Peri‐traumatic factors and PTSD.

Peri‐Trauma				
	Sig.	Exp(B)	95% C.I. for EXP(B)	
			Lower	Upper
Event type	0.802	1.154	0.377	3.528
Age	0.060	1.133	0.995	1.291
Sex	0.354	1.507	0.633	3.591
Exposure type	0.796	0.858	0.271	2.723
Age	0.053	1.139	0.998	1.299
Sex	0.511	1.346	0.555	3.263
Perceived threat to life	0.063	0.394	0.148	1.053
Age	0.037	1.151	1.009	1.313
Sex	0.301	1.592	0.660	3.843
Serious injury to yourself or another	0.150	.434	0.139	1.354
Age	0.052	1.140	0.999	1.300
Sex	0.313	1.568	0.655	3.756
Participants				
Others	0.111			
Oneself	0.036*	3.892	1.092	13.874
Oneself and others	0.318	1.652	0.616	4.429
Age	0.054	1.143	0.998	1.309
Sex	0.224	1.751	0.710	4.318

Table 4 shows PTSD associations with symptomatology; each symptom effect on PTSD was adjusted for sex and age. The odds of PTSD increased with increasing severity of each of the studied symptoms. Furthermore, the associations with PTSD, in a decreasing manner, were associated with dissociative symptoms, followed by symptoms in Group C, Group B, Group E and, finally, Group D.

**TABLE 4:** PTSD association with symptomatology.

	aOR	*p*‐value	95% C.I. for aOR
Group B	1.36	<0.001***	1.19‐1.56
Group C	1.93	<0.001***	1.45‐2.56
Group D	1.29	<0.001***	1.16‐1.44
Group E	1.34	<0.001***	1.17‐1.53
Dissociative symptoms	2.98	0.003**	1.44‐6.16

*Note*: Results are presented as adjusted odds ratios (aORs) and 95% confidence intervals (CIs) for aORs; all models are adjusted for sex and age.

^a^ *p* < 0.05.

**p < 0.01.

***p < 0.001.

Dissociative symptoms were significantly associated with PTSD (aOR = 18.01, 95% CI = [3.06–106.01], *p* = 0.001). When the associations between the presence of dissociative symptoms and the type of event were analyzed, a statistically significant relationship was found for the presence of dissociative symptoms in physical, verbal, and sexual aggression events (Table 5).

**TABLE 5:** Interpersonal violence (physical, verbal, or sexual violence events) and group symptoms.

Peri‐Trauma	
**Symptoms**	***p* value**
Group B	0.015
Group C	0.006
Group D	<.001
Group E	<.001
Dissociative symptoms	0.018

#### 3.5.3 Posttraumatic Factors and PTSD

Subsequent exposure to adverse events was not significantly associated with PTSD (Table 6).

**TABLE 6:** Posttraumatic factors and PTSD.

**Post‐Trauma**				
	**Sig**.	**Exp(B)**	**95% C.I. for EXP(B) **	
			Lower	Upper
Subsequent exposure to adverse events	0.235	1.758	0.693	4.463
Age	0.078	1.125	0.987	1.283
Sex	0.356	1.506	0.632	3.589

## 4 Discussion

### 4.1 Prevalence of PTSD

The first aim of this study was to assess the prevalence of PTSD in a clinical sample of children and adolescents in the Lisbon area. To the best of our knowledge, this is the first study in Portugal that has carried out this analysis using the DSM‐5 criteria. Given that PTSD is traditionally associated with war scenarios and that Portugal is a politically stable country, the aim of this study is to demonstrate that many other events can lead to the development of PTSD, which can occur at any moment in a person's timeline. The studied sample revealed that 35.6% of the children and adolescents met the diagnostic criteria for PTSD. Although the disorder traditionally involves symptoms in the first month after exposure to an event, a subtype with later onset, that is, 6 months after exposure to an event, has also been described (American Psychiatric Association 2013). In this sample, 3% of the cases were included in this group, highlighting the importance of assessing not only the short term but also the long term, as well as the importance of conducting several reassessments over time after diagnosis.

The prevalence described herein is greater than that reported in the literature (Alisic et al. 2014; Mueser and Taub 2008). As this is a clinical sample, a greater degree of risk factors is expected in this psychiatric population, which can potentially lead to the development of PTSD. Therefore, it should be noted that the results obtained highlight a greater number of PTSD diagnoses than those clinically attributed; this may lead us to analyses at least three aspects: first, given that this assessment took into consideration the presence of symptoms at any point in life, the real reason why they were being monitored may not be related; second, PTSD symptomatology may exhibit different characteristics depending on age (and developmental stage), meaning that special attention is essential for this age group; third, we cannot exclude the hypothesis that this disorder is underdiagnosed, as reported in other studies, or often misdiagnosed as major depressive disorder (Thabet and Thabet 2017; Christoffersen and Thorup 2024; Grosso 2009). To assist in this more effective assessment, questionnaires or scales can be used, as is the example of the CAPS‐CA‐5, which has been used worldwide and is already prepared in accordance with the DSM‐5 criteria, which are specific to assessments in children and adolescents.

### 4.2 Pre‐Traumatic Factors and PTSD

Age was the only pre‐traumatic factor that demonstrated a significant association with the development of PTSD in our sample, and it was greater for older participants. These results are in line with those of other studies (Charnsil et al. 2020; El‐Khodary et al. 2020; Yuan et al. 2023). Although the exact mechanism involved is not known, some hypotheses have been proposed: (1) The cause may be related to emotional and biological changes, which can be considered stressful events; (2) adolescence is a developmental period associated with a high risk of exposure to negative or traumatic events and vulnerability; and (3) it may be related to the greater ability to understand the events that occurred and their severity, particularly the concept of death (Charnsil et al. 2020; El‐Khodary et al. 2020; Yuan et al. 2023). As highlighted by Guyer et al. (2016), developmental stages, such as adolescence, are marked by hormonal fluctuations (e.g., estrogen and testosterone) that affect emotional regulation and stress responses. These changes may heighten vulnerability to trauma‐related symptoms like PTSD during adolescence. As individuals age, neurobiological systems also undergo changes, such as a decline in cortisol production, which may influence how trauma is processed. This helps explain why younger individuals may show more acute trauma responses, while adults may experience more chronic symptoms due to the cumulative effects of trauma over time. On the other hand, given that adolescence is often associated with risky behavior, mainly due to the search for challenges and new experiences, it is not surprising that adolescents sometimes put themselves at risk of experiencing traumatic situations. An example, in our sample, was a teenager who had online contact with a person whom she believed to be the same age, and a personal encounter with that person led to sexual abuse.

Importantly, the participants who met the criteria for PTSD earlier in life were just 4 years old, while 20 cases (55.6%) involved children 12 years of age or younger. These data highlight the importance of analyzing exposure to traumatic events throughout life and indicate that PTSD is not only associated with adulthood or war situations, as has been traditionally believed (Mueser and Taub 2008; Havens et al. 2012). Awareness and a high suspicion index are needed to detect PTSD in children, not only in high‐risk contexts but also in low‐risk scenarios, as our results support the underdiagnosis of this disorder.

Although sex, previous exposure to traumatic events, family type, and the Graffar index were not significantly associated with PTSD, the authors believe that these factors should always continue to be part of the routine assessment in mental health care because of the lack of information regarding this disorder and to allow future comparative studies regarding PTSD (Copeland et al. 2007; Pynoos et al. 1999; Fazel et al. 2012).

### 4.3 Peri‐Traumatic Factors and PTSD

With respect to peri‐traumatic factors, after adjusting for sex and age, there were significant associations regarding the type of event and the type of participants involved in the event. With respect to the type of event, it was found that sexual, physical, and verbal violence, that is, interpersonal violence, was associated with a greater risk of developing PTSD, which is in line with what has also been described in other studies (McLaughlin et al. 2013; Lewis et al. 2019). According to epidemiological studies, between 30% and 70% of children who experience physical or sexual abuse develop PTSD (McLaughlin et al. 2013; Lewis et al. 2019). In this regard, it is important to address two issues: (1) Some authors state that these data may reflect the fact that these events are associated with a high degree of a perceived threat to life, which has also been consistently identified as one of the strongest predictors of PTSD; (2) on the other hand, these events often occur with reference figures leading to changes in the attachment process, while deficits in attachment security play a critical role in increasing responses to stress and are associated with the development of PTSD (McLaughlin et al. 2013; Bryant 2023; Ozer et al. 2003). However, it is important to note that other frequently associated risk factors, such as female sex, family dysfunction, low socioeconomic status, and reoccurrence of exposure, if combined, may increase the risk of developing PTSD.

With respect to those involved in the traumatic event, there was a greater association with the development of PTSD when the person was directly involved than when there was the additional involvement of other people (family/friends/strangers) or the involvement of only people other than the person involved. Direct involvement or close proximity has been described as a greater risk for the development of psychiatric disorders, namely, PTSD or prolonged grief disorder (Thierry 2017; Bergman et al. 2017). Therefore, it is important not only to have the skills and tools to address these events but also to have a support network (family, school, community), which has been found to be a great asset for a better clinical outcome and prognosis (Thierry 2017; Bergman et al. 2017). This support has been directly related to greater resilience, which, in turn, has been described as predictive of a lower risk of developing PTSD (Thompson et al. 2018; Feder et al. 2009).

An analysis of the various groups of symptoms of PTSD according to the DSM‐5 criteria revealed an obvious association between all groups and the development of PTSD. However, it is important to highlight that Group C (avoidance) presented a more significant association, followed by Group B (intrusion), Group E (changes in arousal and reactivity), and finally Group D (negative changes in cognitions and mood). These findings can be interpreted from different perspectives. First, it can only be customized to the characteristics of this specific sample; second, we cannot state the hypothesis that the types of events experienced could influence the symptoms present, as has been described in other studies; third, we highlight the fact that Group D symptoms show less association, possibly because of their lower specificity, since several of these symptoms may also occur in a depressive condition and influence the results. Finally, it is important to keep in mind that symptoms may differ in the immediate phases compared with the chronic phases of exposure, meaning that an analysis of the same sample at a different point in time would yield different data (Birkeland et al. 2022; Hoorelbeke et al. 2021; Birkeland and Heir 2017).

Of great importance is also the analysis of the role of dissociative symptoms, a group of symptoms that are not included as diagnostic criteria and that have been regarded as a subtype of PTSD. In our sample, dissociative symptoms were the type of symptoms most significantly associated with the development of PTSD; this highlights dissociative symptoms as a group that should be taken into consideration, not only for clinical assessment but also as a potential diagnostic criterion. If we analyze the moderating effect by sex, it does not exist; however, the probability is greater for males, as described in other studies (Aho et al. 2017; Bennett et al. 2015). The mechanism involved appears to be related to dysfunctional memory processing in trauma, deficient encoding of traumatic memories and later dysfunctional recall and processing of events, as well as a failure to symbolize and integrate traumatic scenes (Peltonen et al. 2017; Ehlers and Clark 2000).

Notably, when the associations between the presence of dissociative symptoms and the types of events were analyzed, a statistically significant relationship was found for the presence of dissociative symptoms in physical, verbal, and sexual aggression events, that is, interpersonal violence. Given that this group of events is most significantly associated with the risk of PTSD, this association with dissociative symptoms becomes more striking and may constitute two very important warning signs. The presence of dissociative symptoms has been associated with greater clinical severity and higher grades of difficulties in recovery, especially in children (when compared with adults), so its assessment and intervention should be the basis for clinicians who encounter these children/adolescents (Peltonen et al. 2017; Sugar and Ford 2012).

### 4.4 Posttraumatic Factors and PTSD

Some posttraumatic factors have been identified as being associated with an increased risk of PTSD, namely, exposure to subsequent adverse events, parental reactions to trauma, coping strategies, and resilience (American Psychiatric Association 2013; Tang et al. 2017). This study restricted the analysis only to exposure to subsequent adverse events. No association was found, not even when adjusted for age and gender. However, the literature supports the association between PTSD and exposure to subsequent adverse events. Hence, preventing or predicting future exposure to adverse events can be an important point of work in reducing the likelihood of developing PTSD.

### 4.5 In the Portuguese Context

In the Portuguese context, these results are consistent with those reported by Pinto et al. (2021), who conducted a two‐wave longitudinal study on Portuguese adolescents. Their research demonstrated that symptoms of depression, anxiety, and PTSD mediated the relationship between childhood trauma and quality of life, highlighting the crucial role of emotional and psychological adjustment in trauma outcomes (Pinto et al. 2021). In addition, the authors identified social support as a key moderating variable, suggesting that supportive interpersonal environments may buffer the negative effects of early adversity—a finding that resonates with patterns observed in the present study (Pinto et al. 2021).

Complementing their longitudinal work, Pinto et al. also validated the Portuguese version of the Child PTSD Symptom Scale (CPSS), providing robust psychometric evidence for the use of this tool in both clinical and research settings (Pinto et al. 2019). This contributes to the methodological foundation for trauma assessment in Portuguese youth, supporting the reliability of self‐reported PTSD symptoms in adolescent populations and aligning with the assessment strategies used in the current study (Pinto et al. 2019).

At the adult level, Cardoso et al. (2020), using data from the World Mental Health Survey Initiative, reported high levels of trauma exposure in the Portuguese population and identified sexual violence—particularly among women—as a powerful predictor of PTSD. Women who reported sexual abuse had a 2.7 times higher likelihood of developing PTSD, even after adjusting for trauma type (Cardoso et al. 2020). This disproportionate impact points to persistent gender‐related vulnerability across the lifespan and underlines the importance of early identification and gender‐sensitive interventions.

While direct comparisons across these studies are limited by methodological differences—namely, the longitudinal design used by Pinto et al. (2021) versus the cross‐sectional approach of Cardoso et al. (2020)—a coherent pattern emerges: early trauma is strongly associated with mental health outcomes, and social factors significantly influence the severity and persistence of symptoms. These findings collectively underscore the need for longitudinal, developmentally‐informed research in Portugal that spans childhood into adulthood, using harmonized assessment tools to capture the complexity and evolution of trauma‐related symptoms over time.

## 5 Strengths and Limitations of the Study

This study has several limitations. First, the data were collected about events that may have occurred at any time in the lives of the participants; as a result, participants may have forgotten some information related to that moment. As this was an analytical cross‐sectional study, longitudinal effects could not be examined, and causal relationships could not be confirmed. Therefore, longitudinal studies are needed to explore the mechanisms underlying these effects. Hence, given the use of a clinical sample, the current findings are not generalizable to other populations, types of trauma, or other contexts/environments. Another limitation of the present study concerns the role of socioeconomic status. Although SES was measured using the Graffar Index and included in the descriptive analysis, it was not incorporated as a predictor variable in the regression models. This decision was based on preliminary analyses indicating limited variability in SES within our sample, which may reflect the characteristics of the population attending public mental health services. Nevertheless, previous research has consistently demonstrated that lower SES is associated with greater exposure to traumatic events and increased vulnerability to PTSD. Therefore, the exclusion of SES from the predictive models limits our ability to fully assess its potential moderating or mediating effects. Future studies with more diverse socioeconomic samples should explore this relationship more comprehensively.

Although studies on population samples provide data with a greater degree of generalizability and take into account that PTSD has been regarded as an underdiagnosed condition, it can also be useful to evaluate this condition in clinical contexts. In our study, we did not assess only one type of predictor but rather pre‐, peri‐, and post‐trauma factors, thus providing a more complete understanding. We consider that the results of the present study have important implications in three main aspects. First, they highlight the importance of continuous screening for PTSD; second, they highlight the importance of improving screening practices, including interdisciplinary efforts to improve the ability of medical service discussions to identify PTSD, many of whom may not have specific knowledge or training in mental health; and third, they highlight the importance of prevention and early intervention efforts to promote child well‐being throughout life.

## 6 Conclusion

The results revealed that 35.6% of the children and adolescents met all the criteria for PTSD. With respect to pre‐traumatic factors, an association was found between the development of PTSD and older participants. With respect to peri‐traumatic factors, there is an association between the development of PTSD and traumatic events of the type of interpersonal violence and those in which one was involved in the event. Dissociative symptoms demonstrated a relationship with the development of PTSD and with the type of event, particularly with more interpersonal violence events. The results of the present study may have implications for a greater understanding of PTSD in this age group, greater knowledge about important warning signs, and the appropriateness of prevention and intervention strategies, thereby allowing us to take the next steps towards personalized medicine, namely, the type of events and type of symptoms experienced.

## Author Contributions

**Inês Barroca**: conceptualization, investigation, writing – original draft, methodology, validation, visualization. **Inês Pinto**: conceptualization, writing – review and editing, supervision, visualization. **Paula Saraiva Carvalho**: conceptualization, supervision, writing—review and editing, visualization.

## Ethics Statement

We affirm that the results of this study have not been published previously and are not currently under consideration elsewhere; the study is original and the author(s)’ own, and no copyright has been breached by the inclusion of any content drawn from another source; the publication has been approved by all co‐authors. Approvals by the Ethics Committees of the hospital entities involved in the study were obtained.

## Clinical Impact Statement

- 35.6% of children and adolescents met all criteria for PTSD.
- Pre, peri‐ and posttraumatic factors can predict the development of PTSD in childhood and adolescence.
- Identifying risk factors and including early assessments by professionals may alert to exposure to traumatic events exposure in childhood and adolescence and can be an is an essential factor strategy in reducing to reduce the number of undiagnosed cases and improving to improve intervention strategies and prognosis in these cases clinical outcomes.

## Conflicts of Interest

The authors declare no conflicts of interest.

## Peer Review

The peer review history for this article is available at https://publons.com/publon/10.1002/brb3.70805.

## Data Availability

The data that support the findings of this study are available on request from the corresponding author. The data are not publicly available due to containing information that could compromise research participant privacy/consent.
